# Unique TLR9 Activation by *Helicobacter pylori* Depends on the *cag* T4SS, But Not on VirD2 Relaxases or VirD4 Coupling Proteins

**DOI:** 10.1007/s00284-022-02813-9

**Published:** 2022-03-03

**Authors:** Nicole Tegtmeyer, Bodo Linz, Yoshio Yamaoka, Steffen Backert

**Affiliations:** 1 Department of Biology, Chair of Microbiology, Friedrich-Alexander-Universität Erlangen-Nürnberg, Staudtstr. 5, 91058 Erlangen, Germany; 2 Department of Environmental and Preventive Medicine, Oita University Faculty of Medicine, Yufu, Japan

## Abstract

The genomes of the gastric bacterial pathogen *Helicobacter pylori* harbor multiple type-IV secretion systems (T4SSs). Here we analyzed components of three T4SSs, the cytotoxin-associated genes (*cag*) T4SS, TFS3 and TFS4. The *cag* T4SS delivers the effector protein CagA and the LPS-metabolite ADP-heptose into gastric epithelial cells, which plays a pivotal role in chronic infection and development of gastric disease. In addition, the *cag* T4SS was reported to facilitate conjugative transport of chromosomal bacterial DNA into the host cell cytoplasm, where injected DNA activates intracellular toll-like receptor 9 (TLR9) and triggers anti-inflammatory signaling. Canonical DNA-delivering T4SSs in a variety of bacteria are composed of 11 VirB proteins (VirB1-11) which assemble and engage VirD2 relaxase and VirD4 coupling proteins that mediate DNA processing and guiding of the covalently bound DNA through the T4SS channel. Nevertheless, the role of the latter components in *H. pylori* is unclear. Here, we utilized isogenic knockout mutants of various *virB* (*virB9* and *virB10*, corresponding to *cagX* and *cagY*), *virD2* (*rlx1* and *rlx2*)*, virD4* (*cag5, traG1/2*) and *xerD* recombinase genes in *H. pylori* laboratory strain P12 and studied their role in TLR9 activation by reporter assays. While inactivation of the structural *cag* T4SS genes *cagX* and *cagY* abolished TLR9 activation, the deletion of *rlx1*, *rlx2, cag5*, *traG* or *xerD* genes had no effect. The latter mutants activated TLR9 similar to wild-type bacteria, suggesting the presence of a unique non-canonical T4SS-dependent mechanism of TLR9 stimulation by *H. pylori* that is not mediated by VirD2, VirD4 and XerD proteins. These findings were confirmed by the analysis of TLR9 activation by *H. pylori* strains of worldwide origin that possess different sets of T4SS genes. The exact mechanism of TLR9 activation should be explored in future studies.

**Supplementary Information:**

The online version contains supplementary material available at 10.1007/s00284-022-02813-9.

## Introduction

*Helicobacter pylori* represents a Gram-negative bacterium that colonizes the human stomach. *H. pylori* infection is common in approximately 50% of the world’s population. While infected patients typically exhibit mild, often asymptomatic chronic gastritis, a subset of patients can develop severe gastric diseases including mucosa-associated lymphoid tissue (MALT) lymphoma, peptic ulceration and gastric adenocarcinoma (reviewed by [1, 2]). Gastric cancer represents an eminent cancer type in humans and comprises about one million new patients annually, accounting for approximately 769,000 deaths in 2020 alone [3]. Owing to these numbers, gastric cancer incidence and mortality rates worldwide rank on position five and four, respectively. The clinical consequences of *H. pylori* infections are affected by numerous key components comprising the host genetic susceptibility, the genotype of the bacteria, various environmental determinants, the diet and microbiota (reviewed by [1, 2]). Host genetic biomarkers, among others, comprise single-nucleotide polymorphisms in various genes coding for multiple cyto- and chemokines (for example IL-1β, IFN-γ, TNF, IL-2, IL-6, IL-8, and IL-10) as well as immune receptors (e.g., NOD1, NOD2, TLR2, TLR4, TLR5, and TLR9) (reviewed by [1, 2, 4]). Studies using animal models such as the Mongolian gerbils pinpointed that *H. pylori* infection and the expression of certain virulence genes are the primary trigger for the development of gastric carcinogenesis (reviewed by [1, 2]).

Worldwide *H. pylori* strains from different continents are genetically highly diverse [5]. During the long co-evolution with its human host, which lasted for over 100,000 years [6], various type-IV secretion systems (T4SSs) were acquired and/or evolved, each of which with specific functions (reviewed by [7, 8]). These T4SSs are molecular transport systems that assemble in the inner and outer membranes of many bacteria and are frequently associated with a protruding pilus [9]. Typically, many canonical T4SSs comprise 11 structural protein subunits, called VirB1 to VirB11 (according to the nomenclature of the prototypical T4SS from *Agrobacterium tumefaciens*), the coupling protein VirD4/TraG and a DNA processing enzyme (relaxase VirD2). In particular, VirB2-VirB11 and VirD4/TraG are obligatory for effector molecule delivery [10]. According to common categorization, the T4SS functions include (i) the transfer of conjugative and other mobile DNA elements, (ii) the release of DNA into the environment, (iii) the uptake of extracellular DNA, (iv) the transport of effector proteins into the supernatant, as well as (v) the direct injection of effector proteins into the cytoplasm of the host cell. In fact, *H. pylori* encodes up to four different T4SSs: (i) the cytotoxin-associated genes T4SS (*cag* T4SS), (ii) the ComB apparatus, (iii) a third T4SS called TFS3 and (iv) a fourth T4SS called TFS4 (reviewed by [8, 11]). The *cag* T4SS mediates the transport of the CagA effector protein into host cells, which is followed by tyrosine phosphorylation by host cell kinases [8, 12]. In addition, ADP-heptose [13], peptidoglycan [14] and chromosomal DNA [15, 16] were also described as substrates translocated into target cells. In contrast, the ComB system facilitates uptake and transport of extracellular DNA into the bacterium [17]. TFS3 as well as TFS4 supposedly represent conjugational DNA transfer systems, which is supported by the discovery of two VirD2-like relaxase enzymes, Rlx1 and Rlx2 [18–20], two VirD4-like coupling proteins (TraG1/2) [18, 19], putative *oriT* (origin of transfer) sequences detected near the TFS3 and TFS4 genes [8, 20], and tyrosine recombinase (XerD) with recognition sites [21]. In addition, TFS3 might deliver the effector protein CtkA, a putative cell-translocating kinase, into target cells [22–24].

The *cag* T4SS, which is present in so-called type-I strains (or *cag*-positive strains), but being absent in type-II isolates (or *cag*-negative strains), contains about 32 genes encoding the described VirB1–VirB11 homologs, VirD4 along with multiple *cag*-specific proteins [25–28]. Electron microscopic experiments visualized the *cag* T4SS core complex, including the Cag3, CagM, CagT, CagX, and CagY proteins [29, 30]. Besides the effects described above, the *cag* T4SS was found to activate integrins [12, 31–34] and two toll-like receptors, TLR5 [35, 36] and TLR9 [15, 16]. Interestingly, TLR9 activation was reported to proceed through translocated chromosomal DNA mediated by the *cag* T4SS, but the exact DNA substrate, *oriT* and mechanism are yet unknown. TLR9 represents an endosome-associated receptor of the innate immune system that detects hypo-methylated cytidine phosphate guanosine (CpG) oligodinucleotides that are typical for bacterial DNA (reviewed in [37–39]). Stimulation of TLR9 appears to occur in a contact-dependent manner between *H. pylori* and host cells, and requires the structural T4SS proteins CagC (VirB2), CagE (VirB3-VirB4), CagL (VirB5), CagT (VirB7), CagX (VirB9), CagY (VirB10), and the Cag-specific components CagM, CagV and Cag3 [15, 16]. However, DNA transfer mediated by canonical T4SSs commonly requires additional factors, including DNA processing enzymes such as VirD2 relaxases and VirD4 (TraG) coupling proteins (reviewed in [9, 10]). Such VirD2 relaxase enzymes are not encoded in the *cag* T4SS, but are present in the TFS3 and TFS4 systems as described above. While these DNA processing enzymes are essential for DNA transfer in a variety of bacteria, their importance in *H. pylori* is unclear. We hypothesized that the VirD2, VirD4/TraG and XerD enzymes might be interchangeable among the various *H. pylori* T4SSs. Thus, in this research we examined the proposed involvement of relaxases Rlx1 and Rlx2 and DNA coupling proteins VirD4 and TraG as well as recombinase XerD in chromosomal DNA transfer-mediated TLR9 activation by *H. pylori*.

## Materials and Methods

### T4SS Genetic Analysis

The genome of *H. pylori* strain P12 (Genbank accession number CP001217.1) was subjected to pairwise BLASTn comparisons against the genomes of strains 26695 (NC_000915.1), N6 (VAPN00000000.1), G27 (NC_011333.1), HPAG1 (NC_008086.1), PMSS1 (CP018823.1), NCTC11637 (NZ_LS483488.1), B128 (NZ_CP024951.1), 7.13 (NZ_CP024953.1), J166 (NZ_CP007603.1), TN2 wt (NZ_AP019730.1), India7 (CP002331.1), Cuz20 (CP002076.1), Shi470 (CP001072.2), Gambia94/24 (CP002332.1), and SouthAfrica7 (NC_017361.1), followed by visual examination in the Artemis Comparison Tool ACT [40]. The nucleotide sequences of the *cag* T4SS (*cag*PAI, about 37 kb), TFS3 region (29 kb) and TFS4 region (40 kb) were extracted from the genome of strain P12. Subsequently performed pairwise tBLASTx comparisons were visualized and analyzed in ACT. Gene synteny was compared to published data [11, 26]. In addition, the inferred protein sequences were extracted and compared in pairwise BLASTp searches, and their inferred function was validated in an NCBI Conserved Domain search.

### Human HEK293-TLR5 Reporter Cells and AGS Cell Line

HEK293 cells (ATCC #CRL-1573) are derived from embryonic kidney. TLR9 activation was studied using HEK293 cells stably transfected with human TLR9/NF-κB/secreted embryonic alkaline phosphatase (SEAP) reporter (HEK-Blue-hTLR9, TLR9^+^). HEK-Blue-Null cells (parental) were used as a negative control (InvivoGen, San Diego/USA; https://www.invivogen.com/hek-blue-htlr9) [15, 16]. These two cell lines were grown in DMEM (Dulbecco’s Modified Eagle Medium) enriched with 110 mg/L sodium pyruvate, 4.5 g/L d-glucose, 4 mM l-glutamine, and 10% fetal calf serum (FCS) (Thermo Fisher Scientific, Massachusetts, USA). AGS cells (ATCC #CRL-1739), a gastric epithelial adenocarcinoma cell line, were cultivated in RPMI-1640 medium supplemented with 10% FCS (Thermo Fisher Scientific, Massachusetts, USA). All three cell lines were subcultured in 75 cm^2^ tissue culture flasks. To all culture media a 1% antimycotic and antibiotic solution was added (Sigma-Aldrich) during long-term propagation. In addition, the HEK293 cell medium contained 10 μg/mL blasticidin (InvivoGen) to maintain the integrated hTLR9 plasmid and 100 mg/mL zeocin to maintain the SEAP plasmid. Before infection, the cells were washed, resuspended in fresh medium without antibiotics, and seeded into 12-well plates (Greiner-Bio-One, Frickenhausen, Germany). Cultivation of cell monolayers was accomplished at 70–80% confluency [41]. In all experiments, the cells were infected with bacteria at a multiplicity of infection (MOI) of 25 for 24 h. The uninfected cells (mock control) were incubated with the same volume of Brain Heart Infusion (BHI) medium.

### Cultivation and Mutagenesis of Bacteria and Infection Experiments

A list of all used strains and isogenic *H. pylori* mutants is provided in Table S1. The mutants were commonly created by insertion of gene cassettes containing a chloramphenicol or kanamycin resistance gene, respectively, using established protocols. Mutants were confirmed by PCR and Sanger sequencing. Where possible, loss of protein expression was confirmed by Western blotting [42, 43]. The individual *H. pylori* strains were grown on GC agar plates enriched with 10% horse serum (PAN-Biotech GmbH, Aidenbach, Germany), trimethoprim (5 μg/mL), nystatin (1 μg/mL) and vancomycin (10 μg/mL). For selection, *H. pylori* mutants were cultivated on media with 8 μg/mL kanamycin or 4 μg/mL chloramphenicol (both from Sigma-Aldrich, St. Louis, USA), respectively. *H. pylori* were grown for 2 days at 37 °C under microaerophilic conditions in AnaeroJars™ (Oxoid, Wesel, Germany) using Oxoid™ CampyGen™ 2.5L gas packs. The bacteria were harvested using sterile cotton swabs and were resuspended in BHI medium.

### SEAP Reporter Gene Assays

The reporter cell line HEK-Blue-hTLR9 (TLR9^+^) and its corresponding parental cell line HEK-Blue-Null1 were infected by *H. pylori* to activate transcription factor NF-κB and alkaline phosphatase-1 (AP-1), which trigger the expression and secretion of secreted embryonic alkaline phosphatase SEAP, which in turn was used as a measure of NF-κB activity [16]. After infection, 20 μL cell culture supernatant (infected vs. non-infected) was mixed with 180 µL Quanti-Blue reagent (InvivoGen) and incubated for 30 min at 37 °C, following the protocol of the manufacturer. The SEAP levels were determined from the OD_620_ values measured with the Infinite F200 Pro microplate reader (Tecan, Grödig, Austria).

### IL-8 ELISA

The concentration of chemokine IL-8 secreted by infected and non-infected HEK-Blue reporter cells was determined using the enzyme-linked immunosorbent assay (ELISA) [44]. To this end, 20 μL of the cell culture supernatant of the above SEAP reporter assay was analyzed using the IL-8 Human Uncoated ELISA Kit (Invitrogen, #88–8086). Supernatants from non-infected samples served as negative control.

### Western Blotting

Infected HEK293 and AGS cell lines were harvested using cell scrapers, and heated at 95 °C for 5 min in 1× Laemmli buffer. Separation of proteins by sodium dodecyl sulfate polyacrylamide gel electrophoresis (SDS-PAGE) was performed on gels with 6–10% polyacrylamide followed by Western blot analysis using ROTI®PVDF membranes (Carl Roth, Karlsruhe, Germany). The membranes were incubated for 1 h at 20 °C with TBS-T buffer (140 mM NaCl, 25 mM Tris–HCl pH 7.4 and 0.1% Tween-20) including either 3% BSA or 5% skim milk [45] to block non-specific binding sites. For detection, the following antibodies were used: mouse monoclonal antibody to GAPDH was obtained from Santa Cruz Biotechnology (Heidelberg, Germany). Phosphorylated and total CagA proteins were identified by successive probing of the blots with the mouse monoclonal α-pan-phosphotyrosine antibody PY99 (Santa Cruz Biotechnology) and rabbit polyclonal antibody against CagA (Austral Biologicals, San Ramon, USA) [46]. Polyvalent horseradish peroxidase (HRP)-coupled secondary goat antibodies were used to detect mouse and rabbit primary antibodies (Thermo Fisher Scientific, Massachusetts, USA). Subsequently, the blots were visualized using the ECL Prime chemiluminescence kit from GE Healthcare as described [47, 48].

### Statistical Tests

All data were obtained from experiments performed in triplicate. GraphPad Prism statistical software (version 8.0) was used for all data analyses. All TLR9 activation data were assessed using One-way analysis of variance (ANOVA) followed by Tukey’s test. The level of statistical significance was determined by using the following *P*-values: *P* ≤ 0.05 (*), *P* ≤ 0.01 (**), *P* ≤ 0.001 (***) and *P* ≤ 0.0001 (****).

## Results

### Genetic Analysis of *H. pylori* T4SSs and DNA Transfer Related Genes

**Fig. 1:**
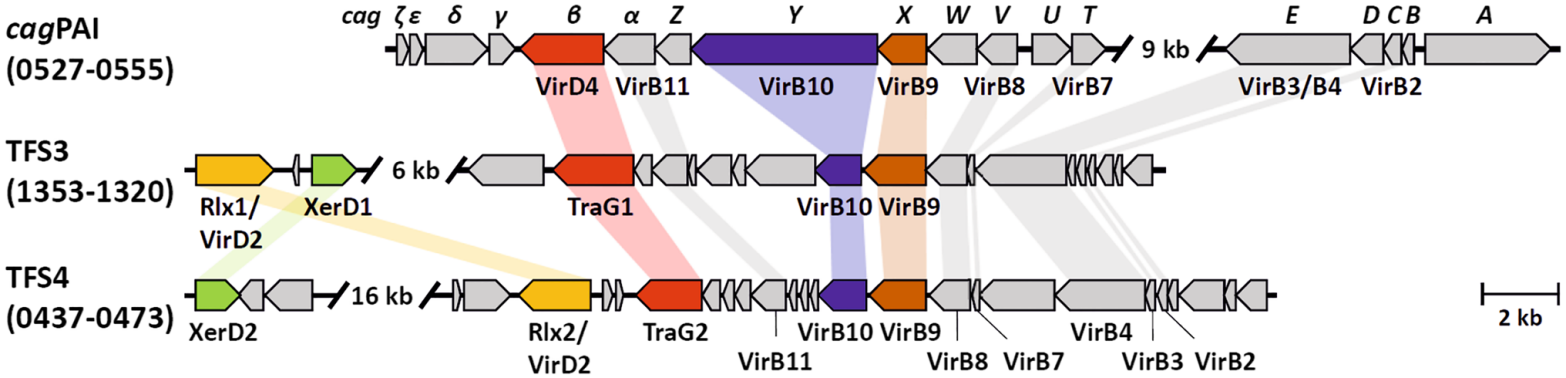
Genetic composition of three T4SSs in *H. pylori* strain P12. Conserved structural components VirB2-VirB4 and VirB7-VirB11 of the T4SSs, as well as DNA coupling proteins TraG/VirD4, DNA relaxases Rlx/VirD2 and recombinase XerD are highlighted. The TFS3 and TFS4 VirB6 proteins are located within the blanked out regions, in the *cag*PAI VirB6 is represented by CagW. The P12 locus tag numbers are indicated next to the T4SS. The fourth *H. pylori* T4SS (the ComB system) was not included in this alignment, because it facilitates the import of free DNA, which was not subject of this study

Pairwise tBLASTx comparisons and BLASTp searches of the extracted protein sequences showed the presence of genes encoding VirB proteins (VirB2-VirB4; VirB6-VirB11) as well as the VirD4 (TraG) coupling protein in each of the three T4SS-encoding genomic fragments, encoding the *cag* T4SS, TFS3 and TFS4 (Fig. 1). In addition to VirB proteins, both TFS3 and TFS4 contained genes coding for two VirD2 relaxases (Rlx1/VirD2 in TFS3, Rlx2/VirD2 in TFS4), two TraG proteins (TraG1 in TFS3 and TraG2 in TFS4) as well as two XerD tyrosine recombinase-like proteins (XerD1 in TFS3 and XerD2 in TFS4). Alignment of three T4SSs revealed that the synteny of the T4SS component genes was conserved, with minor exceptions due to insertion of additional genes in each sequence. In contrast, the *virD2, traG* and *xerD* genes of TFS3 and TFS4 were not present in the *cag*PAI sequence, and order as well as orientation of *virD2* and *xerD* differed among TFS3 and TFS4 (Fig. 1). We also noted that the TFS4-encoded topoisomerase and VirB4 proteins in strain P12, as well as the TFS3-encoded XerD recombinase are likely not functional due to frameshift mutations. The nomenclature of the corresponding genes in strain P12 is shown in Supplemental Fig. S1. The fourth *H. pylori* T4SS, the ComB system, was not included in this study because it is a DNA import apparatus and does not contain DNA export factors such as VirD2, VirD4/TraG and XerD enzymes [8, 17].

### Clinical *H. pylori* Type-I Strains Activate TLR9, But Not Type-II Isolates

**Fig. 2:**
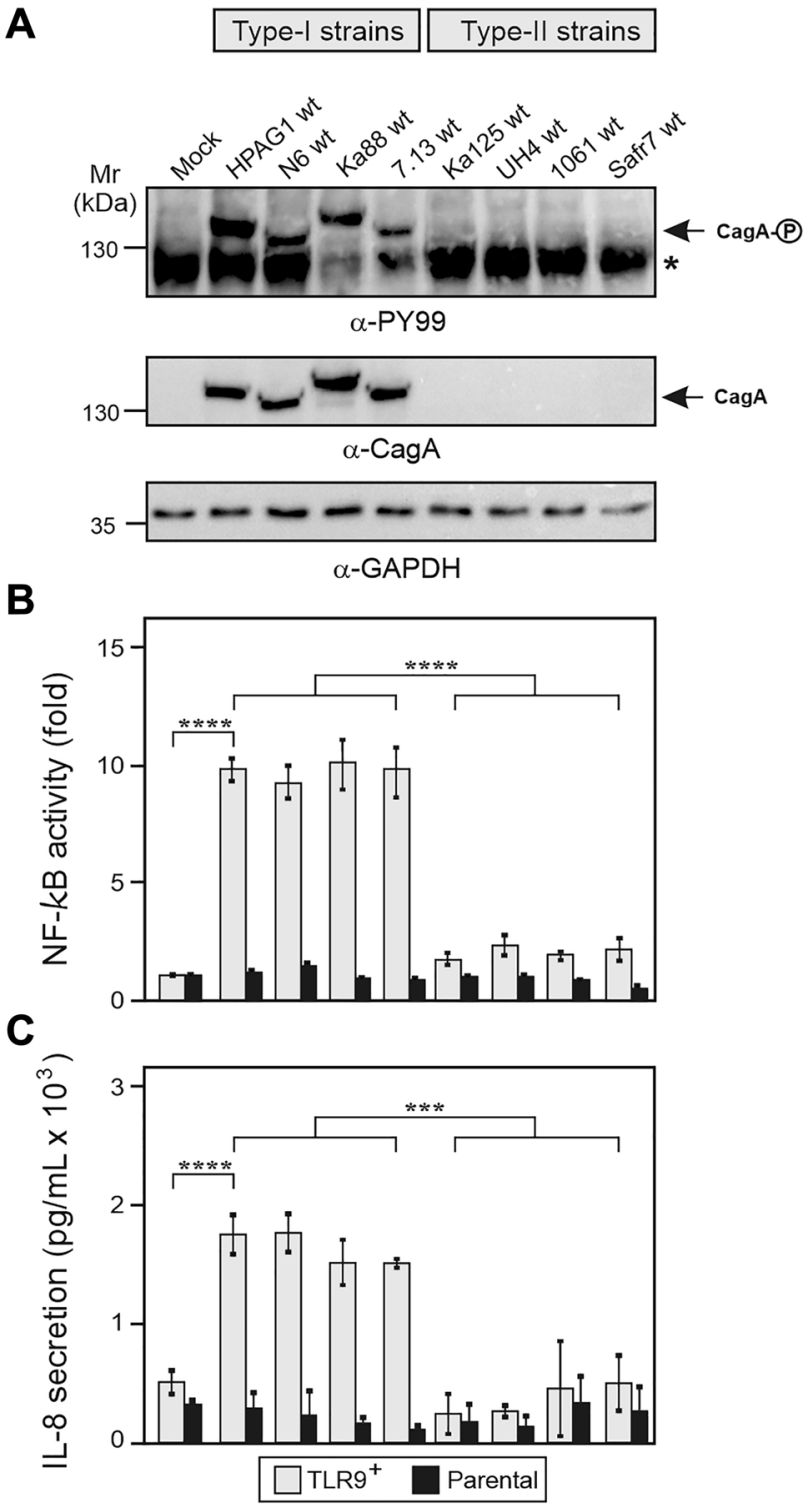
Stimulation of TLR9 activation by T4SS-positive type-I *H. pylori* strains. **A** Infection of AGS cells with various *H. pylori* wt strains. In contrast to type-II strains, type-I *H. pylori* isolates express a functional T4SS that enables injection and subsequent phosphorylation of CagA in the cytoplasm. CagA phosphorylation and expression was monitored by Western blot analysis of corresponding protein lysates using antibodies against PY99 and CagA. The asterisk in panel **A** indicates an unidentified phosphorylated 125 kDa protein of the host cell. GADPH was used as loading control. **B** Quantification of TLR9 activation by the SEAP reporter assay. **C** IL-8 secretion measured by ELISA. Quantitative data are presented as means ± SD. Safr7: SouthAfrica7

To investigate if a collection of different clinical *H. pylori* type-I (*cag*PAI-positive) and type-II (*cag*PAI-negative) isolates can activate TLR9, we performed infections of the HEK293 epithelial reporter cell system. Parallel infection of gastric epithelial AGS cells confirmed that type-I (but not type-II) *H. pylori* strains express a functional *cag* T4SS and exhibit comparable CagA expression and phosphorylation levels (Figs. 2A, S2). The HEK293 parental control wild-type (wt) cells that were previously reported to be naturally devoid of expressing most of the known innate immune receptors such as TLRs, thus reducing background NF-ҡB activation by *H. pylori* to a minimum [49], were used in the corresponding reporter assays. The HEK293 cells were stably transfected with two constructs, human TLR9 (TLR9^+^) and SEAP (secreted embryonic alkaline phosphatase), which allowed to monitor the activity of NF-κB, a major downstream pro-inflammatory transcription factor [15, 16]. Our results showed that clinical *cag* T4SS-positive strains including HPAG1 (Sweden), N6 (France), Ka88 (Germany) and 7.13 (USA) activated NF-κB in TLR9^+^ cells 8- to 11-fold in comparison to the parental SEAP control cells without TLR9 (Fig. 2B). In contrast, *cag* T4SS-negative isolates including Ka125 (Germany), UH4 (Germany), 1061 (Australia) and SouthAfrica7 (Safr7, South Africa) failed to activate TLR9, showing only baseline NF-κB levels (Fig. 2B). NF-κB activation via TLR9 correlated with elevated secretion of chemokine IL-8 in the same experiments (Fig. 2C). These results confirm that the *cag*PAI is required for profound TLR9 stimulation by *H. pylori*.

### TLR9 Activation by *H. pylori* Depends on the *cag*PAI, But Not on Known T4SS Effector Molecules

**Fig. 3:**
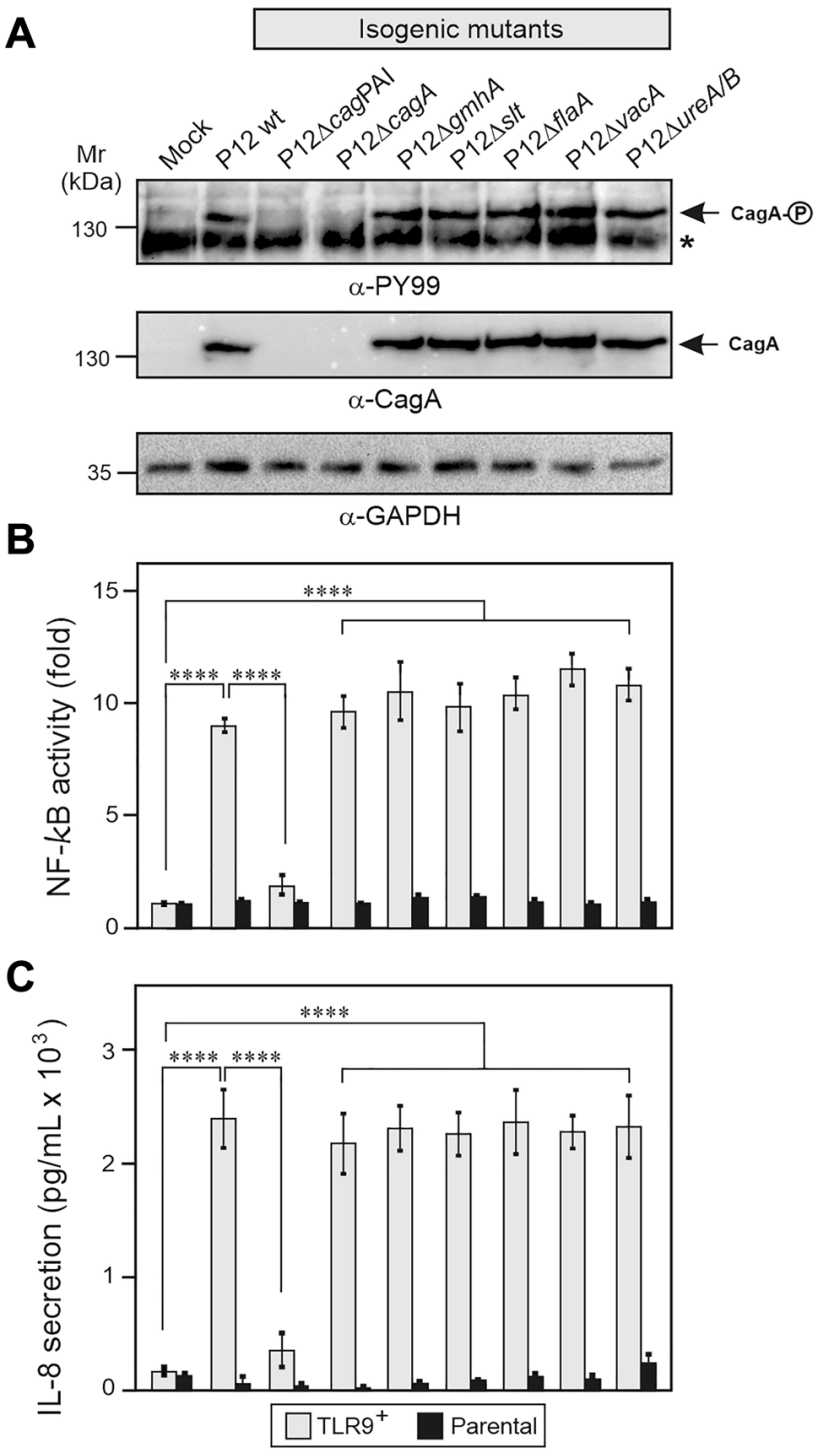
TLR9 activation by *H. pylori* requires the *cag*PAI, but not CagA, ADP-heptose or peptidoglycan effector molecules. **A** Infection of AGS cells with the parental wt strain P12 and isogenic mutants. Western blots of corresponding protein lysates were analyzed with the antibodies shown in the panel. α-PY99: phosphorylated CagA; α-CagA: total CagA; α-GAPDH: loading control. The asterisk in panel **A** indicates an unidentified phosphorylated 125 kDa protein of the host cell. **B** Quantification of TLR9 activation by the SEAP reporter assay. **C** IL-8 secretion measured by ELISA. Quantitative data are presented as means ± SD

In the following experiments, these TLR9 reporter cells were infected with multiple isogenic mutants of well-established *H. pylori* virulence factors in comparison to the parental wt control (Table S1). As expected, infection of the gastric AGS cells revealed proper CagA expression and phosphorylation (Figs. 3A, S3). While Δ*flaA,* Δ*vacA*, and Δ*ureA/B* deletion mutants did not affect CagA phosphorylation and TLR9 activation, deletion of the entire *cag*PAI abolished these responses (Fig. 3B). Interestingly, *H. pylori* deletion mutants Δ*cagA*, Δ*gmhA* and Δ*slt* defective in genes encoding known translocated T4SS effector molecules CagA (Δ*cagA*), ADP-heptose (Δ*gmhA*) and peptidoglycan (Δ*slt*) also activated CagA phosphorylation (except Δ*cagA*), TLR9 stimulation (Fig. 3B), and IL-8 secretion (Fig. 3C) like wt *H. pylori.* Hence, TLR9 activation through the *cag* T4SS is not coupled to injection of these known effector molecules.

### *Helicobacter pylori-*Mediated TLR9 Activation Does Not Require VirD2 Relaxases, VirD4 Coupling Proteins and XerD Recombinase

**Fig. 4:**
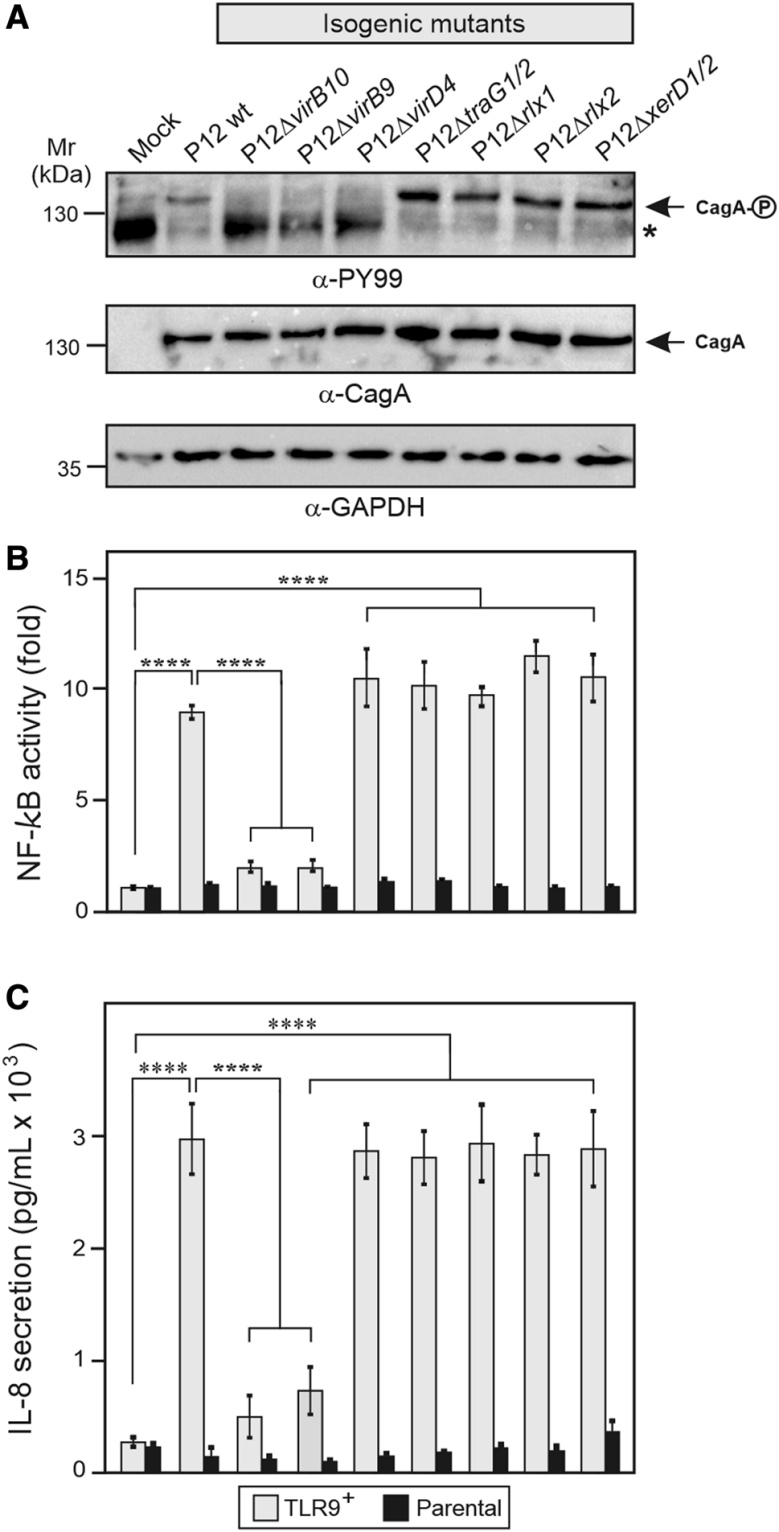
TLR9 activation by *H. pylori* does not involve VirD2 relaxases, VirD4 coupling proteins or XerD recombinase. **A** Infection of AGS cells with the parental wt strain P12 and isogenic mutants. Western blots of corresponding protein lysates were analyzed with the antibodies shown in the panel. α-PY99: phosphorylated CagA; α-CagA: total CagA; α-GAPDH: loading control. The asterisk in panel **A** indicates an unidentified phosphorylated 125 kDa protein of the host cell. **B** Quantification of TLR9 activation by the SEAP reporter assay. **C** IL-8 secretion measured by ELISA. Quantitative data are shown as means ± SD

Next, we generated isogenic deletion mutants of structural *cag* T4SS component genes, Δ*virB9* and Δ*virB10*, of *virD2* relaxases (Δ*rlx1* and Δ*rlx2*)*,* and of *virD4* coupling factors (Δ*virD4* of the *cag* T4SS and Δ*traG* TFS3/TFS4 double mutant) of *H. pylori* strain P12 (Table S1), and investigated their importance in TLR9 activation by SEAP reporter assays. Parallel infection of the gastric AGS cells showed proper CagA expression and phosphorylation by a subset of strains as expected (Figs. 4A, S4). Interestingly, while inactivation of the structural *cag* T4SS genes *virB9* and *virB10* abolished CagA phosphorylation and TLR9 activation, inactivation of *rlx1*, *rlx2* or *traG1/2* did not affect CagA phosphorylation and activated TLR9 (Fig. 4B) and IL-8 secretion (Fig. 4C) like wt *H. pylori*. An exception is *virD4,* which is required for CagA phosphorylation and the corresponding mutant activated TLR9 like wt *H. pylori* (Fig. 4B). Given that the TFS4 genetic island is capable of self-excision and can be transferred between strains by a conjugation-like process facilitated by a XerD tyrosine recombinase [21], we also analyzed an isogenic *xerD* knockout mutant (∆*xerD2*) in TFS4. Note, that *xerD1* in TFS3 (locus_tag BPP12_1351) is likely not functional due to a frameshift mutation (Table 1). However, this Δ*xerD2* knockout strain also stimulated CagA phosphorylation and TLR9 activation during infection (Fig. 4A, B). Thus, our results indicate that all previously reported *H. pylori* relaxases and coupling proteins as well as XerD recombinases that are involved in DNA transfer by canonical T4SSs play no role in TLR9 activation by *H. pylori*.

**Table 1:** Presence or absence of T4SS genes encoding coupling protein TraG, relaxase, recombinase/integrase XerD, and *cag*PAI in worldwide *H. pylori* strains in relation to TLR9 activation

Strain	Locus							
	TFS3			TFS4			*cag* T4SS	TLR9 activation
	1337^a^	1353^a^	1351^a^	0454^a^	0451^a^	0437^a^	0527-0555^a^	
	*traG1*	*rlx1*	*xerD1*	*traG2*	*rlx2*	*xerD2*	*cag*PAI	
Europe								
P12	+	+	+^b^	+	+	+	+	+
26695	−	+^c^	+	+^c^	+^b^	−	+	+
N6	−	+^d^	+	+	+	+	+	+
G27	−	−	−	+	+	−	+	+
HPAG1	−	−	−	−	−	−	+	+
Australia								
PMSS1	−	−	−	−	−	−	+	+
NCTC11637	−	+^b^	+	+	+^b^	+	+	+
USA								
B128	+	+^b^	+	−	+	+	+	+
7.13	+	+^b^	+	−	+	+	+	+
J166	+	+	+	−	−	−	+	+
Asia								
TN2	−	+^b^	+	+	+	+	+	+
India7	+	+	+	−	+^b^	+	+	+
Peru								
Cuz20	−	−	−	+	+	−	+	+
Shi470	−	−	−	+	+	−	+	+
Africa								
Gambia94/24	+^a^	+	+	+	+^a^	+	+	+
SouthAfrica7	−	−	−	+	+	+	−	−

+ present, − absent

^a^Locus_tag (HPP12_*) in genome of P12 strain (Genbank accession number CP001217.1)

^b^Not functional due to frameshift mutation

^c^Not functional due to insertion of an IS element

^d^Not functional due to in-frame stop codon

### TLR9 Activation and Role of T4SS Genes During Infection with Worldwide Clinical *H. pylori* Strains

The results obtained above with isogenic T4SS mutants of strain P12 were surprising. To corroborate these findings, HEK-Blue-hTLR9 reporter cells were infected with several clinical wt *H. pylori* isolates of worldwide origin, for which full genome sequences are available (Table 1). The results show that all *cag* T4SS-positive strains from Gambia (Gam94/24), Germany (P12), Italy (G27), France (N6), Sweden (HPAG1), India (India7), Japan (TN2wt), Australia (NCTC11637, PMSS1), USA (B128, 7.13, J166) and Peru (Sat464, Shi470) activated TLR9, but not the *cag* T4SS-negative strain SouthAfrica7. Interestingly, while all of the above TLR9-activating strains possess the known *cag* T4SS *virB/D* genes, pairwise genome comparisons revealed that the TFS3 and TFS4 genes are present in some but not all strains (Table 1). For example, strain 7.13 carries *traG1* and *xerD1* (TFS3) as well as *rlx2* and *xerD2* genes (TFS4), but *rlx1* (TFS3) and *traG2* (TFS4) are missing. In G27, the TFS3 genes are entirely missing and *xerD2* is absent in TFS4. In 26695, the *traG* genes are either missing (*traG1*, TFS3) or non-functional due to insertion of an IS element (*traG2*, TFS4). Most remarkably, while strains PMSS1 and HPAG1 do not possess any of the analyzed TFS3 and TFS4 genes, the two strains strongly activated TLR9 (Table 1). Thus, these data confirmed that the proposed DNA transfer enzymes VirD2 (Rlx1 and Rlx2), TraG (TraG1 and TraG2) and XerD (XerD1 and XerD2) encoded on the TFS3 and TFS4 gene clusters play no role in TLR9 activation by *H. pylori*.

## Discussion

TLRs represent an important group of pathogen recognition receptors (PRRs), which detect microbial structures that are evolutionarily conserved (reviewed in [37–39, 50]). For a long time, *H. pylori* was thought to avoid immune recognition by TLRs, mainly due to the poor stimulatory activity of its lipopolysaccharide (LPS) and flagellin (reviewed in [49, 51–53]). However, more recent studies indicated that *H. pylori* activates two important TLRs, TLR5 [35, 36] and TLR9 [15, 16], in a T4SS-dependent manner, that facilitate the detection of *H. pylori* by its host [35, 52–54]. While TLR5 is stimulated by the interaction with two structural elements of the *cag* T4SS pilus, CagL and CagY, TLR9 has been reported to be activated by translocated chromosomal *H. pylori* DNA as shown by fluorescence microscopy in HEK293-TLR9 reporter cells [16]. TLR9 represents an intracellular PRR, which is predominantly expressed in the cytoplasm of macrophages, dendritic cells and specific antigen-presenting cells as well as cancer cells (reviewed in [38, 39]). The primary ligand of TLR9 is hypo-methylated CpG DNA originating from microbes, and this interaction commonly triggers inflammatory signal transduction events via type-I interferons and pro-inflammatory cytokines produced through transcription factor NF-ҡB, which finally results in the engulfment and killing of intruding pathogens (reviewed in [38, 39]). Remarkably, *H. pylori-*infected Tlr9^−/−^ knockout mice obtained significantly stronger inflammation scores in comparison to infected wt mice [15]. At the molecular level, gastric mucosal titers of IL-17 were largely upregulated in infected Tlr9^−/−^ knockout mice compared with infected wt mice, but not typical T_H_1 or T_H_2 cytokines [15]. In agreement with these observations, infection of IL-17A^−/−^ knockout mice by *H. pylori* resulted in reduced levels of gastritis compared to wt mice. All these activities in mice were clearly *cag*PAI-dependent. Together, it appears that *H. pylori* employs its *cag* T4SS and TLR9 to modulate the host immune responses, probably to ensure persistent infection [51]. On the other hand, TLR9 may also be involved in cancer development, as various reports demonstrated that TLR9 activity is associated with enhanced malignancy, and it impacts assorted immune activities against cancer (reviewed in [38, 39]). However, the underlying mechanisms that define the role of TLR9 in the onset and progression of *H. pylori-*triggered gastric cancer are not entirely understood and need to be unraveled in further studies.

Conjugation in bacteria represents T4SS-mediated transfer of DNA between many bacterial species, and also facilitates delivery of DNA from bacteria into eukaryotic target cells upon infection [9, 10]. Canonical T4SSs involved in this conjugational DNA transfer typically consist of two major elements, the DNA transfer and replication system (called Dtr) and the mating pair formation system (named Mpf), following the characteristic “replication and secretion” scheme (reviewed in [7, 9, 10, 55, 56]). The Mpf system is basically composed of the above described 11 membrane-spanning VirB or Tra proteins, which translocate substrates across the membranes and form an extracellular pilus [9, 10]. In addition, the Dtr comprises the initiation complex of conjugative DNA transfer, called the relaxosome. The driving factor is the relaxase or VirD2 protein, which is commonly loaded onto the origin of transfer (*oriT*) by accessory DNA-binding proteins [57]. The VirD2 enzyme (relaxase) then cleaves a phosphodiester bond at the nicking site in one strand of the *oriT*, and covalently binds to the 5’end of the cleaved DNA strand. Subsequently, the single-stranded DNA is translocated, which is mediated by an adapted rolling-circle replication mechanism [57]. Thus, the substrate DNA of conjugative T4SSs is always exported as a DNA–protein complex of the relaxase VirD2 with single-stranded DNA, but not as naked DNA. After the circle of transfer is completed, the process is terminated by the cleaving-joining activity of the relaxase. Another critical factor for conjugative DNA transfer is the so-called coupling protein, shown to bridge the Dtr with the Mpf by specific interactions. The best investigated coupling factors are VirD4 (from the T-DNA transfer system of the tumor inducing (Ti) plasmid in *A. tumefaciens*) and TraG (from the IncP1 plasmid RP4) (reviewed in [9, 10, 55, 56]). These proteins exhibit NTPase activity and were reported to form a pore via oligomerization and bind DNA without sequence specificity. Remarkably, *H. pylori* encodes a VirD4 ortholog in the *cag* T4SS, and two TraG-like proteins as well as two VirD2 relaxase proteins in TFS3 and TFS4, respectively (Fig. 1). We therefore proposed that these enzymes may be interchangeable among the T4SSs and could be involved in the proposed *H. pylori* DNA transfer and TLR9 activation by the *cag*PAI. However, while knockout of the structural *cag* T4SS genes *virB9* and *virB10* abolished TLR9 activation by *H. pylori*, in good agreement with previous studies [15, 16], inactivation of *rlx1*, *rlx2, cag5* and *traG1/2* did not affect the activation of TLR9. These deletion mutants activated TLR9 similar to their parental wt bacteria. In addition, knockout of *xerD* genes in TFS3 and TFS4, encoding a tyrosine recombinase with reported function in horizontal DNA transfer by a conjugation-like mechanism between *H. pylori* strains [21], is also not involved in TLR9 activation by *H. pylori*. Thus, we are obviously facing a remarkable paradox. Our present findings therefore suggest the presence of a previously unknown T4SS-dependent mechanism of TLR9 activation by *H. pylori*. Since a conjugative T4SS-dependent DNA export without involvement of a relaxasome and a DNA coupling factor is so far unknown, the exact mechanism of DNA transfer by *H. pylori* and involved DNA processing/guiding enzymes etc. still remain mysterious, and need to be studied in detail in future research.

## Conclusion

 The *H. pylori cag* T4SS was previously reported to play a role in conjugative transfer of chromosomal bacterial DNA into the host cell cytoplasm, where the injected *H. pylori* DNA activates TLR9 [15, 16, 51, 52, 54]. Canonical DNA-delivering T4SSs in numerous bacteria are comprised of structural VirB proteins, VirD2 relaxase as well as VirD4/TraG coupling proteins that mediate DNA processing and guiding of the covalently bound DNA through the T4SS channel. However, our data show that neither of the previously suspected proteins VirD2, TraG, or recombinase XerD are involved in TLR9 activation by *H. pylori*. Thus, our data suggest the presence of a novel, unique non-canonical *cag* T4SS-dependent mechanism of TLR9 activation by *H. pylori* that is independent of VirD2, TraG and XerD proteins.

## Supplementary Information

Below is the link to the electronic supplementary material.

Supplementary file1 (TIF 3666 KB)

Supplementary file2 (TIF 3217 KB)

Supplementary file3 (TIF 3459 KB)

Supplementary file4 (TIF 3233 KB)

Supplementary file5 (DOCX 56 KB)

## Data Availability

All data are available.
