# Supplementary material for: Unique TLR9 Activation by Helicobacter pylori Depends on the cag T4SS, But Not on VirD2 Relaxases or VirD4 Coupling Proteins
Source: Curr Microbiol. 2022 Mar 3;79(4):121. doi: 10.1007/s00284-022-02813-9 (PMC8894178; doi:10.1007/s00284-022-02813-9)
Supplement: Supplementary file 5 — Supplementary file5 (DOCX 56 KB) [file 284_2022_2813_MOESM5_ESM.docx]

**Table S1. *Helicobacter pylori* strains used in this study.**

| **Strain name** | **Geographic origin** | **Mutated gene(s)**^a^ | **Affected T4SS(s)** | **Antibiotic**  **Resistance**^c^ | **Reference/**  **Origin** |
| --- | --- | --- | --- | --- | --- |
| Gambia94/24wt | The Gambia | none | none | none | Dr. Douglas Berg |
| SouthAfrica7 wt | South Africa | none | none | none | Dr. Douglas Berg |
| HPAG1 wt | Sweden | none | none | none | Dr. Lars Engstrand |
| N6 wt | France | none | none | none | Dr. Agnes Labigne |
| B128 wt | USA | none | none | none | Dr. Richard Peek jr. |
| 7.13 wt | USA | none | none | none | Dr. Richard Peek jr. |
| Ka88 wt | Germany | none | none | none | [1] |
| Ka125 wt | Germany | none | none | none | [1] |
| UH4 wt | Germany | none | none | none | [1] |
| 1061 wt | Australia | none | none | none | [2] |
| G27 wt | Italy | none | none | none | Dr. Antonello Covacci |
| 26695 wt | UK | none | none | none | ATCC 700392D-5 |
| TN2 wt | Japan | none | none | none | [3] |
| India7 wt | India | none | none | none | Dr. Douglas Berg |
| J166 wt | USA | none | none | none | [4] |
| NCTC11637 wt | Australia | none | none | none | Dr. Richard Peek |
| PMSS1 wt | Australia | none | none | none | Dr. Adrian Lee |
| Cuz20 wt | Peru | none | none | none | Dr. Douglas Berg |
| Shi470 wt | Peru | none | none | none | [5] |
| P12 wt | Germany | none | none | none | [6] |
| P12∆*cag*PAI |  | HPP12_0527-0555 | Cag | Kan^R^ | [7] |
| P12∆*gmhA* |  | HPP12_0857 | Cag | Cam^R^ | [8] |
| P12∆*slt* |  | HPP12_0658 | Cag | Cam^R^ | [9] |
| P12∆*cagA* |  | HPP12_0555 | Cag | Cam^R^ | [10] |
| P12∆*flaA* |  | HPP12_0609 | none | Cam^R^ | [10] |
| P12∆*vacA* |  | HPP12_0884 | none | Cam^R^ | [10] |
| P12∆*virB9* |  | HPP12_0535 | Cag | Cam^R^ | [10] |
| P12∆*virB10* |  | HPP12_0534 | Cag | Cam^R^ | [10] |
| P12∆*virD4* |  | HPP12_0531 | Cag | Cam^R^ | [7] |
| P12∆*traG1/2* |  | HPP12_1337  HPP12_0454 | TFS3/TFS4^b^ | Cam^R^, Kan^R^ | [7] |
| P12∆*rlx1* |  | HPP12_1353 | TFS4 | Cam^R^ | [7] |
| P12∆*rlx2* |  | HPP12_0451 | TFS3 | Cam^R^ | [7] |
| P12∆*xerD2* |  | HPP12_0437 | TFS4^b^ | Cam^R^ | [11] |

^a^ locus_tag in genome of strain P12 (Genbank accession CP001217.1)

^b^ a second *xerD* gene (HPP12_1351) in TFS3 is mutated and inactive in strain P12

^c^ abbreviations: Cam^R^, chloramphenicol resistance; Kan^R^, kanamycin resistance

**Supplementary References**

1. Backert S, Schwarz T, Miehlke S, Kirsch C, Sommer C, Kwok T, Gerhard M, Goebel UB, Lehn N, Koenig W, Meyer TF (2004) Functional analysis of the *cag* pathogenicity island in *Helicobacter pylori* isolates from patients with gastritis, peptic ulcer, and gastric cancer. Infect Immun 72:1043-56

2. Goodwin A, Kersulyte D, Sisson G, Veldhuyzen van Zanten SJ, Berg DE, Hoffman PS (1998) Metronidazole resistance in *Helicobacter pylori* is due to null mutations in a gene (*rdxA*) that encodes an oxygen-insensitive NADPH nitroreductase. Mol Microbiol 28:383-93

3. Suzuki R, Satou K, Shiroma A, Shimoji M, Teruya K, Matsumoto T, Akada J, Hirano T, Yamaoka Y (2019) Genome-wide mutation analysis of *Helicobacter pylori* after inoculation to Mongolian gerbils. Gut Pathog 11:45

4. Linz B, Windsor HM, McGraw JJ, Hansen LM, Gajewski JP, Tomsho LP, Hake CM, Solnick JV, Schuster SC, Marshall BJ (2014) A mutation burst during the acute phase of *Helicobacter pylori* infection in humans and rhesus macaques. Nat Commun 5:4165

5. Kersulyte D, Kalia A, Gilman RH, Mendez M, Herrera P, Cabrera L, Velapatino B, Balqui J, Paredes Puente de la Vega F, Rodriguez Ulloa CA, Cok J, Hooper CC, Dailide G, Tamma S, Berg DE (2010) *Helicobacter pylori* from Peruvian amerindians: traces of human migrations in strains from remote Amazon, and genome sequence of an Amerind strain. PLoS One 5:e15076

6. Backert S, Ziska E, Brinkmann V, Zimny-Arndt U, Fauconnier A, Jungblut PR, Naumann M, Meyer TF (2000) Translocation of the *Helicobacter pylori* CagA protein in gastric epithelial cells by a type IV secretion apparatus. Cell Microbiol 2:155-64

7. Backert S, Kwok T, Konig W (2005) Conjugative plasmid DNA transfer in *Helicobacter pylori* mediated by chromosomally encoded relaxase and TraG-like proteins. Microbiology (Reading) 151:3493-503

8. Maubach G, Lim MCC, Sokolova O, Backert S, Meyer TF, Naumann M (2021) TIFA has dual functions in *Helicobacter pylori*-induced classical and alternative NF-kappaB pathways. EMBO Rep 22:e52878

9. Tegtmeyer N, Neddermann M, Lind J, Pachathundikandi SK, Sharafutdinov I, Gutierrez-Escobar AJ, Bronstrup M, Tegge W, Hong M, Rohde M, Delahay RM, Vieth M, Sticht H, Backert S (2020) Toll-like Receptor 5 Activation by the CagY Repeat Domains of *Helicobacter pylori*. Cell Rep 32:108159

10. Pachathundikandi SK, Tegtmeyer N, Arnold IC, Lind J, Neddermann M, Falkeis-Veits C, Chattopadhyay S, Bronstrup M, Tegge W, Hong M, Sticht H, Vieth M, Muller A, Backert S (2019) T4SS-dependent TLR5 activation by *Helicobacter pylori* infection. Nat Commun 10:5717

11. Fischer W, Windhager L, Rohrer S, Zeiller M, Karnholz A, Hoffmann R, Zimmer R, Haas R (2010) Strain-specific genes of *Helicobacter pylori*: genome evolution driven by a novel type IV secretion system and genomic island transfer. Nucleic Acids Res 38:6089-101
